# Supplementary material for: ApisTox: a new benchmark dataset for the classification of small molecules toxicity on honey bees
Source: Sci Data. 2025 Jan 2;12:5. doi: 10.1038/s41597-024-04232-w (PMC11696378; doi:10.1038/s41597-024-04232-w)
Supplement: Supplementary file 1 — Supplementary information [file 41597_2024_4232_MOESM1_ESM.pdf]

ApisTox - supplementary information

	Herbicides		Fungicides		Insecticides		Other agrochemicals		Unknown	
**Split type**	Train	Test	Train	Test	Train	Test	Train	Test	Train	Test
Stratified random	35%	32%	18%	22%	22%	22%	21%	21%	6%	4%
Time	32%	41%	19%	21%	24%	15%	22%	16%	5%	7%
MaxMin	37%	21%	18%	21%	22%	20%	18%	33%	5%	6%

Table 1. Pesticide type distributions for different splits.

Note that pesticides can have more than one type, so it is possible for a given split type for train or test to sum up to more than 100%.
